# A simple one-step procedure to synthesise gold nanostars in concentrated aqueous surfactant solutions†

**DOI:** 10.1039/c9ra02384d

**Published:** 2019-07-30

**Authors:** Ferenc Liebig, Ricky Henning, Radwan M. Sarhan, Claudia Prietzel, Clemens N. Z. Schmitt, Matias Bargheer, Joachim Koetz

**Affiliations:** University of Potsdam, Institute for Chemistry 14476 Potsdam Germany koetz@uni-potsdam.de; Institute for Physics 14476 Potsdam Germany; Cairo University, Chemistry Department, Faculty of Science Cairo 12613 Egypt; Humboldt-Universität zu Berlin, School of Analytical Sciences Adlershof (SALSA) 10099 Berlin Germany; Max Planck Institute of Colloids and Interfaces 14476 Potsdam Germany

## Abstract

Due to the enhanced electromagnetic field at the tips of metal nanoparticles, the spiked structure of gold nanostars (AuNSs) is promising for surface-enhanced Raman scattering (SERS). Therefore, the challenge is the synthesis of well designed particles with sharp tips. The influence of different surfactants, *i.e.*, dioctyl sodium sulfosuccinate (AOT), sodium dodecyl sulfate (SDS), and benzylhexadecyldimethylammonium chloride (BDAC), as well as the combination of surfactant mixtures on the formation of nanostars in the presence of Ag^+^ ions and ascorbic acid was investigated. By varying the amount of BDAC in mixed micelles the core/spike-shell morphology of the resulting AuNSs can be tuned from small cores to large ones with sharp and large spikes. The concomitant red-shift in the absorption toward the NIR region without losing the SERS enhancement enables their use for biological applications and for time-resolved spectroscopic studies of chemical reactions, which require a permanent supply with a fresh and homogeneous solution. HRTEM micrographs and energy-dispersive X-ray (EDX) experiments allow us to verify the mechanism of nanostar formation according to the silver underpotential deposition on the spike surface in combination with micelle adsorption.

## Abstract 2

Due to the enhanced electromagnetic field at the tips of metal nanoparticles, the spiked structure of gold nanostars (AuNSs) is promising for surface-enhanced Raman scattering (SERS).

## Graphical abstract

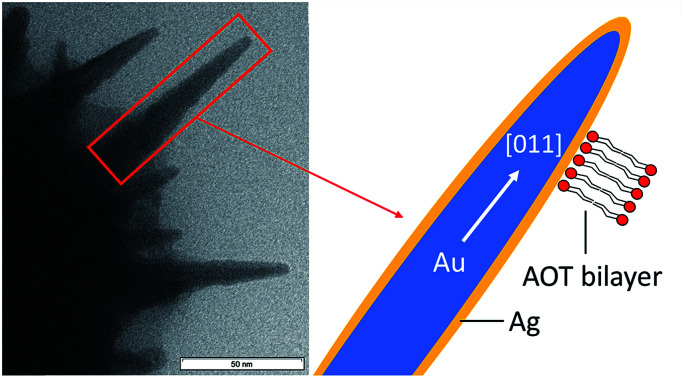

## 1. Introduction

As a result of their special chemical and physical properties, the attention given to gold nanoparticles (AuNPs) expanded with the enhanced potential of their applications in catalysis, photonics, biological sensing, and nanomedicine.^1–8^ Therefore, the shape- and size dependent plasmon resonance of the particles and the related enhanced electromagnetic field is of special interest in electroanalysis and surface enhanced Raman scattering (SERS).^9–15^

The extraordinary role of sharp tips and spikes in surface enhanced Raman scattering leads to the synthesis of star-shaped nanoparticles, *i.e.*, nanostars and nanoflowers, with well-defined tips and spikes.^16–24^ In general protocols of synthesis of gold nanostars (AuNSs) can be divided into seed mediated methods and one-pot procedures.^25^ Therefore, different capping molecules have been used, *e.g.*, cetyltrimethylammonium bromide (CTAB) or chloride (CTAC), sodium dodecyl sulfate (SDS), polyvinylpyrrolidone (PVP), polydiallyldimethylammonium chloride (PDADMAC), 2-[4-(2-hydroxyethyl)-1-piperazinyl] ethane-sulfonic acid (HEPES) and gelatin. For example Wu *et al.* have shown a seeded-growth method in the presence of CTAB and silver cations,^26^ and Li *et al.* a procedure to obtain AuNSs with tunable morphology in the presence of the polycation PDADMAC.^27^ A seed mediated route using HEPES as capping agent was used by other authors.^28^ However, most methods are performed in the presence of CTAB or PVP. Wang *et al.* have shown that sheet-like nanoflowers are formed under UV light irradiation of an aqueous gold chloride solution in the presence of polyvinylpyrrolidone (PVP) and silver ions. Thus, PVP and Ag^+^ ions served as structure-directing agents.^17^ For making AuNSs, PVP-coated gold seeds in ethanol were added rapidly to a mixed concentrated dimethylformamide (DMF) solution containing HAuCl_4_ and PVP.^18^ A modified protocol enabled Khoury and Vo-Dinh to tune the UV absorption of the nanostars between 725 and 850 nm.^19^ A one-pot environmentally benign route was shown by Jena and Raj using HEPES as a reducing and stabilizing agent.^20^ Later the same authors used 5-hydroxyindole-3-acetic acid to obtain gold nanoflowers in a seed- and surfactant-less synthesis.^21^ A citrate-based gold seed solution can be used in a two-step procedure for making monodisperse gold nanostars by adding silver nitrate and ascorbic acid (AA).^22^ Other authors used ascorbic acid from star-fruit juice as reducing component for getting gold nanoflowers by a green method in absence of surfactants.^23^

By adding ascorbic acid to gold clusters, incorporated into catanionic surfactant-based vesicles, nanoflowers can be synthesized, too.^24^ In that case interactions between the anionic surfactant dioctyl sodium sulfosuccinate (AOT) and the cationic surfactant CTAB are of special relevance for the seed mediated gold cluster formation, and the final nanoflower formation. Inspired by these results and our molecular dynamics (MD) simulations about AOT micelle and bilayer adsorption on gold surfaces,^29,30^ we started to investigate the formation of gold nanoparticles in presence of mixed surfactants. As anionic surfactants we have used AOT and sodium dodecyl sulfate (SDS), and as cationic surfactant benzylhexadecyldimethylammonium chloride (BDAC), due to the fact that AOT and BDAC build up mixed micelles, already shown experimentally as well as by MD simulations.^31^

Herein, we report a successful synthesis of well-designed gold nanostars in concentrated AOT and SDS solutions in absence and presence of the cationic surfactant BDAC. We tune the morphology of the AuNSs and the corresponding UV absorption between 550 and 1100 nm *via* the BDAC concentration and show SERS enhancement factors up to 1.5 × 10^4^ measured in solution.

## 2. Experimental details

### 2.1. Materials

Tetrachloroauric acid trihydrate (HAuCl_4_·3H_2_0) was purchased from VWR. l(+)-Ascorbic acid (AA) and benzylhexadecyldimethylammonium chloride (BDAC) were obtained from Carl Roth. Silver nitrate AgNO_3_, sodium dodecyl sulfate (SDS) and dioctyl sodium sulfosuccinate (AOT) with a purity of 98% has been acquired from Sigma Aldrich. For all experiments Milli-Q Reference A+ water was used.

### 2.2. Synthesis of gold nanostars

The AuNSs were prepared in different surfactant template phases based on AOT or SDS in absence or presence of defined amounts of the cationic surfactant BDAC. A 0.04 or 0.16 M micellar AOT solution was mixed with 1.5 ml of a 2 mM HAuCl_4_ and 80 μl of a 2 mM AgNO_3_ solution. To initiate the reduction process 80 μl of 0.1 M ascorbic acid were added. In case of SDS, the surfactant concentration was fixed at 0.025 and 0.1 M, respectively. The parameters of the other compounds remain unchanged. The catanionic micelles were produced by mixing the original AOT as well as SDS micellar solutions with equivalent amounts of micellar BDAC solutions in the concentration range between 0.001 and 0.01 M.

### 2.3. Preparation of SERS substrates

For SERS measurements, 10 μl of 10 mM ethanolic solution of the 4-nitrothiophenol (4-NTP) were mixed with 1 mL of the gold nanostars for 1 hour. 500 μl were subsequently injected into a closed glass vial and further capped with paraffin film to prevent the evaporation of the solution during the measurements.

### 2.4. Methods

The UV-vis absorption spectra were recorded with a Shimadzu UV-2600 spectrophotometer in the wavelength range between 200 and 1400 nm. For the determination of the gold nanoparticle morphology, the transmission electron microscope JEM-1011 (JEOL) at an acceleration voltage of 80 kV as well as the JEM-2200FS (JEOL) at 200 kV for high resolution (HRTEM) and fast Fourier transformation (FFT) were used. The EDX experiments were recorded with a JED-2300 (JEOL) EDX-detector at the JEM-2200FS. Samples were dropped on carbon-coated copper grids and rapidly dried by removing the excess liquid with filter paper. For the morphological characterization at least 50 particles were analysed according to the particle size, core diameter and spike length. Dynamic light scattering (DLS) and zeta potential measurements were performed by using the Malvern Nano Zetasizer 3600.

The Raman spectra were recorded using a confocal alpha 300 Raman microscope from WITec equipped with an excitation laser at 785 nm wavelength. The laser beam was focused onto the solution in the glass vial through 20× microscope objective. The spectra were measured with an integration time of 10 seconds with a thermoelectrically cooled CCD detector DU401A-BV from Andor placed behind the UHTS 300 spectrometer (WITec) with a spectral resolution of 3 cm^−1^. The Raman band of a silicon wafer at 520 cm^−1^ was used to calibrate the spectrometer.

## 3. Results and discussion

### 3.1. Formation of gold nanostars

By adding silver nitrate and ascorbic acid at room temperature to a highly diluted aqueous gold chloride solution in absence of surfactants a violet blue dispersion with an absorption peak at 590 nm can be obtained after few seconds. Herein, AgNO_3_ was used as a shape-directed and ascorbic acid as reducing agent. After a short time period, the Au nanoparticles agglomerate and precipitate, leading to a colorless solution.

**Fig. 1:**
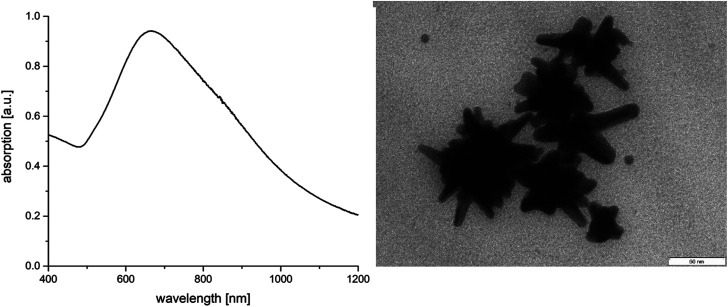
Absorption spectra of AOT stabilized AuNSs and corresponding TEM micrograph.

When the same experiment is performed in a turbid 0.16 M AOT solution or a 0.1 M SDS solution, two orders above the critical micellization concentration (CMC) of the surfactants, *i.e.*, 2.5 mM for AOT^32^ and 8 mM for SDS,^33^ a quite different behavior is observed. In presence of AOT micelles and lamellar liquid crystals^34^ in the template phase a red-shift of the UV maximum from 590 nm to 670 nm can be observed in UV-vis experiments (Fig. 1). The corresponding TEM micrographs show AuNSs with diameter of about 74 nm and well-designed spikes with a length of 24 ± 5 nm (compare Fig. 1 and Table 1). For a more detailed characterization of the nanostar morphology we have analyzed the core diameter and spike length as well as the core/spike ratio in comparison to the data shown by Liz-Marzan^18^ and Khoury *et al.*^19^ A characteristic feature of the AuNSs synthesized in the concentrated AOT template phase is the core/spike ratio of about 1.5 : 1.

**Table 1:** TEM analysis of AuNSs stabilized by AOT and BDAC

Sample	BDAC conc. [M]	Size [nm]	Core [nm]	Spike length [nm]	Core/spike ratio
0	—	74 ± 13	36 ± 10	24 ± 5	1.5 : 1
A	0.001	73 ± 8	41 ± 7	18 ± 5	2.3 : 1
B	0.005	95 ± 18	48 ± 13	22 ± 7	2.2 : 1
C	0.01	101 ± 15	75 ± 12	24 ± 5	3.1 : 1
D	0.05	196 ± 30	158 ± 28	40 ± 16	3.9 : 1

The negative zeta potential of −47 mV implies the presence of AOT molecules on the nanostar surface, which are responsible for the long-term stability of the dispersion. Recently, we have shown by MD simulations that AOT micelles and AOT bilayers strongly adsorb on gold surfaces resulting in distorted cylindrical micelles or more rigid bilayers attached to the {111} facets.^29,30^ Therefore, we can conclude that AuNSs stabilized by AOT micelles and/or bilayers are formed.

It was obvious that the quality of the spikes differentiates from rounded to sharp tips. To obtain high enhancement signals in SERS, the spikes should be well designed and sharpened. Furthermore, the particles should be ideally monodisperse in order to assign the signal increase to the shape of the spikes.

To compare the above results, SDS as another anionic surfactant with sulfur groups was used.

The particle morphology in presence of SDS micelles is analyzed in comparison to the AOT system (compare Table 2 and Fig. S1†). The average size is almost the same, and the UV absorption maximum is in the same region at 660 nm. The spikes are somewhat smaller, and therefore, the core/spike ratio somewhat higher with 1.8 : 1. The zeta potential is −41 mV and thus comparable to the AOT stabilized particles indicating a SDS bilayer on the particle surface.

**Table 2:** TEM analysis of AuNSs stabilized by SDS and BDAC

Sample	BDAC conc. [M]	Size [nm]	Core [nm]	Spike length [nm]	Core/spike ratio
0	—	69 ± 18	34 ± 12	19 ± 8	1.8 : 1
A	0.001	56 ± 15	37 ± 13	13 ± 8	2.8 : 1
B	0.005	75 ± 24	48 ± 18	15 ± 4	3.2 : 1
C	0.01	120 ± 50	84 ± 37	20 ± 4	4.2 : 1
D	0.05	160 ± 41	99 ± 28	26 ± 5	3.8 : 1

A decrease of the surfactant concentration (0.04 M AOT, 0.025 M SDS) leads to the same particle morphology, and therefore, to the same absorption behavior. Recently, we have shown that CTAB-AOT-mixed catanionic vesicles can be used as a template phase for making gold nanoflowers in a two-step process.^24^ The corresponding UV-vis spectrum of the resulting nanoflowers with an inner dense packed platelet structure looks quite similar to the absorption spectra shown here in Fig. 1 and S1.†

This leads us to the open question, if we can tune the nanostar morphology – and the UV absorption and SERS performance – by adding a cationic surfactant, like BDAC?

For this reason, BDAC was used in combination with both anionic surfactants to improve the AuNS structure toward more and sharper spikes. In contrast to the single surfactant synthesis, a change of the BDAC concentration and thus a change in the AOT/BDAC ratio leads to a change in the morphology as well as in the absorption maximum.

**Fig. 2:**
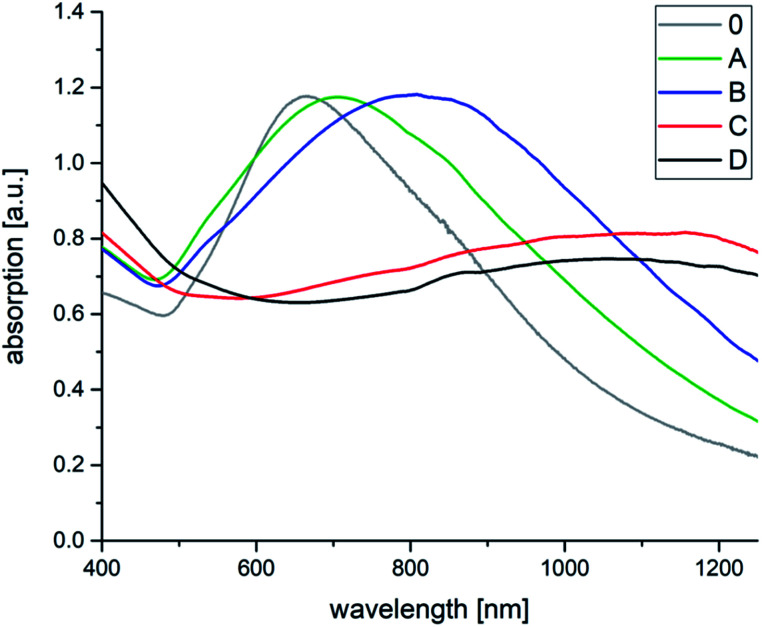
Absorption spectra of AOT–BDAC stabilized AuNSs without BDAC (0) and with BDAC concentration of (A) 0.001 M, (B) 0.005 M, (C) 0.01 M, (D) 0.05 M.

**Fig. 3:**
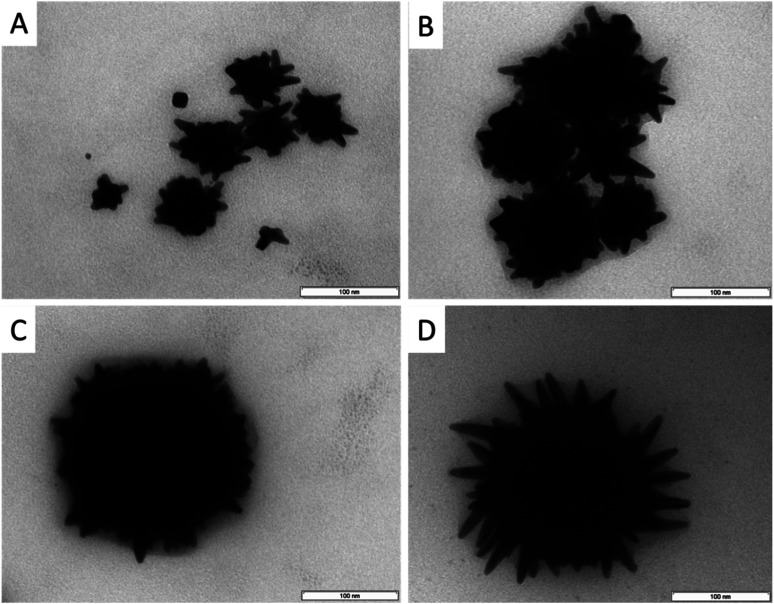
TEM micrographs of AOT–BDAC stabilized AuNSs at different BDAC concentration. (A) 0.001 M, (B) 0.005 M, (C) 0.01 M, (D) 0.05 M.

As can be seen in Fig. 2, the increasing BDAC concentration leads to a strong red-shift in the absorption spectrum from 700 nm to 1200 nm. Comparing the absorption curves with data from the TEM analysis (Table 1, Fig. 3 and S2†), the shift can be related to an increase of the core size and the core/spike ratio. At the lowest BDAC concentration of 0.001 M (sample A), the core size was around 41 nm and showed a similar morphology to the AOT-based AuNSs meaning only few rounded spikes. At a BDAC concentration of 0.005 M the absorption maximum is shifted to 800 nm without a significant change of the core/spike ratio. By increasing the BDAC concentration to 0.01 M a different morphology can be observed (compare also the TEM micrographs at lower magnification in Fig. S2†). On the one hand, the size of the AuNSs core is increased, and on the other hand the core/spike ratio becomes significant larger (>3). For this reason, the particles have a more sphere-like appearance with a flat and broad absorption range > 1000 nm. At a BDAC concentration of 0.05 M, a redoubling of the core size of the AuNSs is performed, but the tips are elongated and sharpened. Under these conditions, AuNSs with a core/spike ratio of 3.9 : 1 and a similar broad absorption range above 1000 nm were produced. The zeta potential of −68 mV illustrates the high stability of the particles. One can conclude that the UV absorption is mainly influenced by the core size and the core/spike ratio. With increasing core size and core/spike ratio a strong blue shift is observed.

**Fig. 4:**
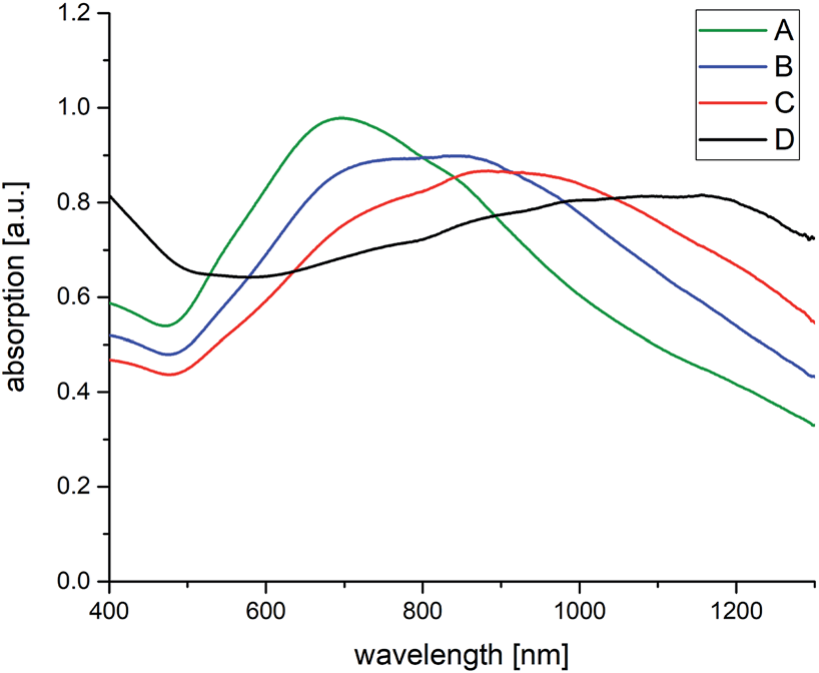
Absorption spectra of SDS–BDAC stabilized AuNSs with BDAC concentration of (A) 0.001 M, (B) 0.005 M, (C) 0.01 M, (D) 0.05 M.

**Fig. 5:**
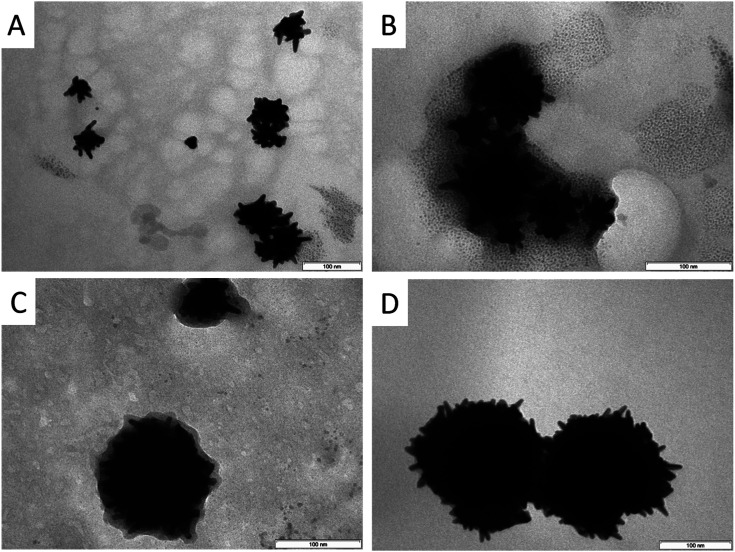
TEM micrographs of SDS–BDAC stabilized AuNSs at different BDAC concentration. (A) 0.001 M, (B) 0.005 M, (C) 0.01 M, (D) 0.05 M.

Spectroscopic (compare Fig. 4) and TEM investigations (compare Table 2 and Fig. 5) of the SDS-based system in presence of different amounts of BDAC reveal a stepwise increase of the core size accompanied by an increase of the core/spike ratio. However, the core/spike ratio is significant larger compared with AOT, only at the highest BDAC concentration the core/spike ratio is quite similar for AOT and SDS. The UV absorption range is shifted to higher wave length with increasing BDAC concentration and becomes more unspecified and broader because of a higher polydispersity, revealed by a higher error range in size distribution. Therefore, one can conclude that the red shift is mainly influenced by the core size, and the broadness of the peak by the size distribution of the particles and the core/spike ratio, which is significantly larger in presence of SDS.

The main difference in the morphology between SDS–BDAC and AOT–BDAC systems is related to the length and sharpness of the spikes (Tables 1 and 2). That means in presence of SDS significantly smaller and shorter spikes are formed with a higher core/spike ratio. However, the number of spikes is comparable, but the polydispersity of the particles is higher. Therefore, the following experiments were focused on the AOT–BDAC system. In comparison to the single surfactant system an increasing concentration of BDAC leads to larger particles with a higher number of better defined spikes. Furthermore, it can be seen in the TEM analysis that without BDAC the ratio between core and spikes is lower, meaning longer spikes and smaller cores.

To clarify the role of the liquid crystals in comparison to the micelles we have decreased the AOT concentration to 0.04 M, *i.e.* below the solubility limit, where only micelles exist. For the absorption and stability, no influence of the AOT concentration can be observed, meaning the same morphology of the particles.

### 3.2. Mechanism of AuNS formation

**Fig. 6:**
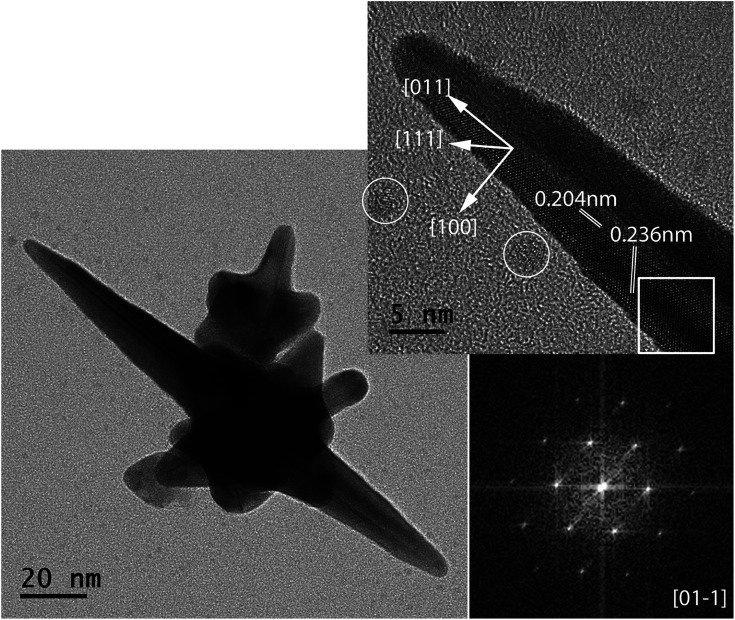
Single nanostar TEM micrograph including HRTEM of the spike with corresponding FFT pattern of the marked square area.

To understand the mechanism of AuNS formation, HRTEM investigations were performed. The micrograph in Fig. 6 shows a single long spike with twin planes at the lowest BDAC concentration of 0.001 M. Another example of a high resolution TEM micrograph of an AuNS is shown in Fig. S3.† The corresponding FFT pattern demonstrates a growth in the [011] direction. This indicates a similar crystallization as observed by Kumar *et al.*^18^ with PVP as symmetry breaking component. In the vicinity of the spikes, individual gold and silver clusters can be found. Therefore, one can conclude that gold clusters embedded in the micellar template phase start to grow up to AuNSs. Preferred nucleation processes at (111) and (100) facets^35^ seem to be responsible for the spike formation.

Yuan *et al.*^22^ have already pointed out that for the formation of gold nanostars Ag^+^ ions are required in analogy to the silver-assisted growth of gold nanorods, already discussed in more detail by Catherine Murphy.^36,37^ Guyot-Sionnest^38^ described a mechanism of Ag^0^ underpotential deposition on the growing gold nanorods. This mechanism was identified on the basis of EDAX measurements^36^ and ICP atomic emission experiments.^37^ However, EDAX and ICP cannot distinguish between Ag^0^ and Ag^+^ and the final distribution of silver within the nanorods. Recently, Tebbe *et al.* show a silver-overgrowth of gold nanorods in presence of the cationic surfactant BDAC, demonstrating the important role of surfactants in that process.^39^

**Fig. 7:**
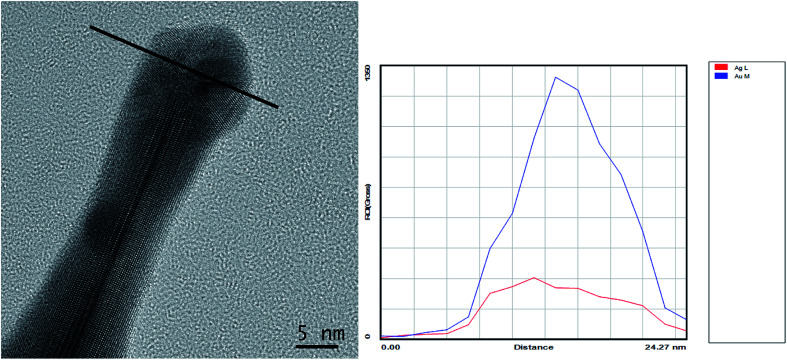
HRTEM micrograph of a gold nanostar-spike with the corresponding EDX-line scan across the spike.

Inspired by these mechanistic considerations, we have performed EDX measurements. Fig. 7 shows an EDX-line scan for the elements silver and gold across the spike of a nanostar. The gold content is strongly increased from both sides reaching a maximal value in the middle of the spike, as expected for a rod-like structure. The curve of the silver content is much flatter, which can be explained by a silver shell surrounding the gold rod. To the best of our knowledge this is a first experimental proof of the existence of a silver layer surrounding the spikes. To underline this statement we have investigated several AuNSs by EDX analysis with a similar result (compare Fig. S4 in the ESI† part).

Recently, we have shown by MD simulations that AOT molecules, AOT micelles as well as AOT bilayers can bind selectively to {111} facets of gold.^29,30^ In presence of AOT and BDAC mixed AOT–BDAC micelles are formed which adsorb at {111} gold facets, too.^31^ Furthermore, MD simulations have shown that NaCl formed by the corresponding counterions, *i.e.*, Na^+^ and Cl^−^, is directly adsorbed at the gold surface, which is of special relevance for the crystallization process. That means AOT and especially AOT–BDAC micelles served as structure-directing agents in similarity to Ag^+^ (ref. 36–40) hinder the growth in vertical direction, and stabilize the resulting nanostars. In conclusion, AuNSs are formed in the micellar AOT–BDAC template phase by growing up of individual spikes in 〈011〉 direction at the periphery of preliminary formed gold nanoparticle cores. The core size, spike shape and length can be tuned by varying the BDAC content in the template phase. AOT surfactants as well as AOT micelles adsorb on the gold surface,^29^ and stabilize very small AuNSs with long spikes and hinder the growth to larger particle dimensions. In presence of BDAC mixed micelles were formed^31^ with a significant lower tendency to adsorb at the gold surface. Therefore, the growth process is less hindered resulting in larger nanoparticles with an increased core radius. Already at very low BDAC concentrations (sample A) in the mixture (molar ratio BDAC : AOT = 1 : 160) the influence of the mixed micelles is identified *via* a shift of the UV maximum to higher wavelength. At a molar ratio BDAC : AOT = 1 : 3 (sample D) the mixed micelles become much more dominant resulting in large AuNSs with diameter of about 200 nm with long spikes. The increased zeta potential of −68 mV can be related to a second AOT bilayer sheet, already proposed by our MD simulations.^30^

### 3.3. SERS performance and investigations of plasmon-driven reactions

Metal nanoparticles act as efficient antennas for electromagnetic radiation from near infrared to UV frequencies. The captured energy can either be reradiated or internally converted into energetic charge carriers (hot electron/hole pair) and subsequently heat.^41^ The radiation scattering from these small nanoobjects leads to a very high local electromagnetic field in the near-field close to the particle surface. This strong electric field can significantly amplify a variety of optical signals such as Raman scattering, where it is revived as surface-enhanced Raman scattering (SERS).^42^ The enhancement is particularly strong in areas called “hot spots”, *e.g.*, in nano-sized gaps between the plasmonic particles or at nano-tips with high aspect ratios. Therefore, SERS experiments are often performed on solid substrates, where the hot spots are generated from the aggregation of the particles or engineered by lithography means. On the contrary, several applications require the measurements to be performed in an aqueous solution. For example, the ultrafast spectroscopy requires a fresh sample spot each measurement since the ultrafast laser could melt the particles.^43^ Photothermal cancer therapy and bioimaging require the injection of the sample into biological fluids.^44^ Herein, we use SERS technique to show the performance of the prepared gold nanostars dispersed in an aqueous solution. The thiolated molecules such as the 4-nitrothiophenol molecules (4-NTP) are commonly used as a model probe in SERS application owing to their high affinity to the metal particles. We investigate an aqueous particle solution mixed with 0.1 mM of the 4-NTP in a closed glass vial to avoid its evaporation during the light irradiation.

**Fig. 8:**
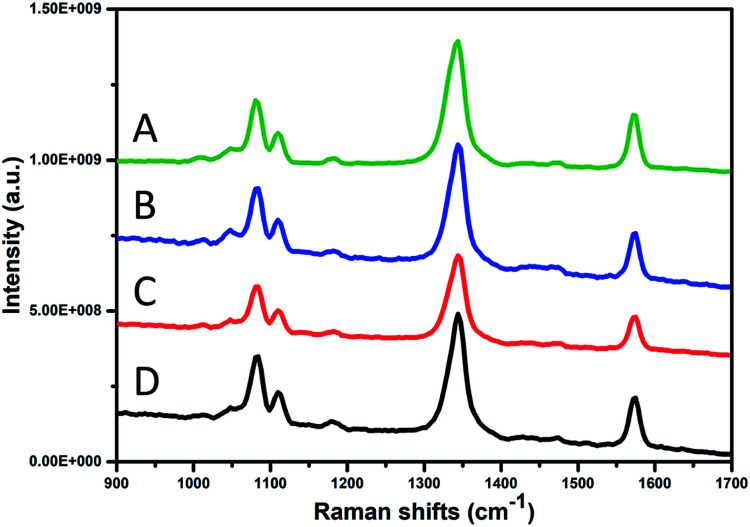
SERS performance of the Au stars prepared with BDAC concentration of (A) 0.001 M, (B) 0.005 M, (C) 0.01 M, (D) 0.05 M.

Fig. 8 shows the SERS performance of the gold nanostars prepared with different BDAC concentrations. The SERS spectra are dominated with the main three Raman peaks of the 4-NTP at 1077, 1332, and 1575 cm^−1^, assigned to the C–H bending, NO_2_, and the C=C stretching modes, respectively.^45,46^ Surprisingly, despite their off-resonance excitation, the gold nanostars prepared with the highest BDAC concentration (black spectrum) produced approximately the same signal enhancement as those prepared with lowest BDAC concentration (green spectrum). This can be attributed to their well-defined structures with many sharp tips on their surface resembling the shape of the sea urchin.^47^ It is known that these sharp tips serve as nanoantennas enhancing the electromagnetic field and therefore, the Raman scattering.^48,49^ We believe that these structures should produce even better signal enhancement at their resonance conditions,^50^ which could be of more importance for biological applications (*e.g.*, photothermal therapy and bioimaging). This also explains the somewhat lower signal intensity of sample C.

**Fig. 9:**
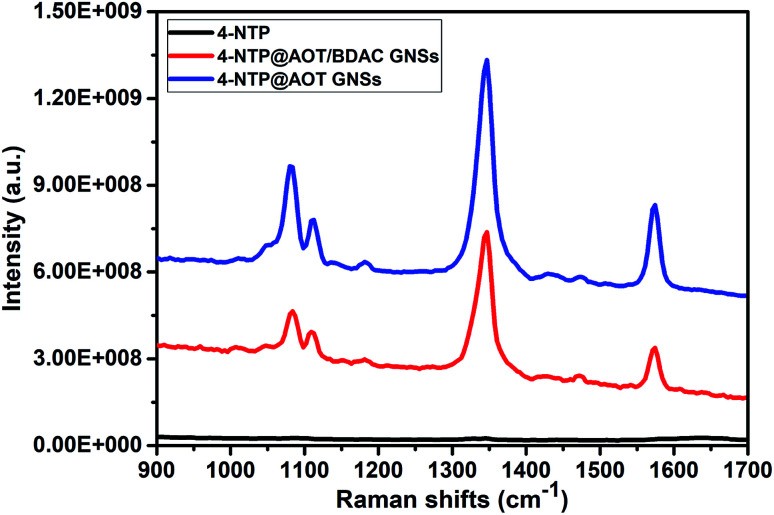
Raman spectra of 4-NTP molecules dissolved in aqueous solution in presence (blue and red spectra) and in absence (black) of the gold nanostars.

Fig. 9 compares the SERS spectra of the gold with the Raman spectrum of 4-NTP at a ten time higher concentration (10 mM) as shown in Fig. 8. Both types of the gold nanostars displayed a well-resolved Raman signature of the 4-NTP, whereas the Raman spectrum of 4-NTP without the gold stars displayed a very weak signal showing the direct influence of SERS. To give an estimation of the enhancement factor, the intensity of the strongest vibrational mode (NO_2_) of the 4-NTP is further compared according to the following formula;
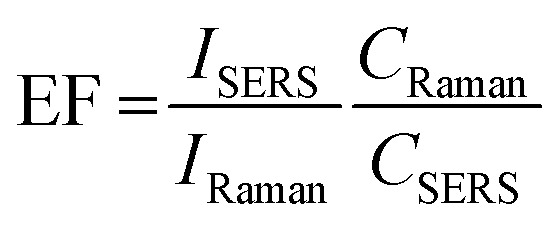
where *I*_SERS_ and *I*_Raman_ are the intensities of the NO_2_ vibrational mode (vibrational mode at 1332 cm^−1^) of the 4-NTP molecule in presence and absence of the gold nanostars, respectively. While *C*_SERS_ and *C*_Raman_ are the concentrations of the 4-NTP solution in presence and absence of the gold particles, respectively. The enhancement factors were estimated to be 1.5 × 10^4^ and 9.5 × 10^3^ for sample A and D of the AOT/BDAC-coated gold nanostars, respectively. Generally, the goldnanostars of sample D are shown to be good candidates for the signal enhancement owing to their long nano-tips. However, the difference in the signal enhancement could be attributed to their plasmon resonance, where the AuNSs of sample A exhibit a plasmon band located more in resonance with the excitation wavelength as compared to sample D. The SERS signal seems to be stable and reproducible over several measurements. The reproducibility of the spectra was confirmed by refreshing the solution several times before each measurement, confirming the homogeneity of the hot spots as well as the dispersity of the particles.

However, we further decreased the concentration of the analyte 4-NTP molecule (10^−6^ M) to unfold the detection capacity of our gold nanostars. Interestingly, the Raman signature of 4-NTP have been clearly observed in both types of the gold nanostars even at a shorter integration time of one second (compare Fig. S4†). This might confirm the enhancement effect of the gold nanostars probing low concentration of the analyte at short integration time. Therefore, we believe that our gold nanostars might be useful for systematic studies that require SERS in a liquid environment, especially for ultrafast studies of chemical reactions using the pump probe technique.^41^

## 4. Conclusions

In this work, we present a one-step synthesis of gold nanostars with a high reproducibility in micellar AOT template phases in absence and presence BDAC. AOT micelles and bilayers stabilize individual gold nanostars, and hinder the growth to larger dimensions. At the periphery of the colloidal gold nanoparticles individual spikes are formed. The spike crystallization is preferred in 〈011〉 direction by Ag underpotential deposition and a selective binding of AOT micelles and bilayers to {111} facets at the spike sides. The core size and spike length can be tuned by adding the cationic surfactant BDAC. In that case mixed micelles were formed having a lower affinity to adsorb at the gold surface. In dependence on the AOT/BDAC ratio it becomes possible to produce well-structured core/spike-shell nanostars with defined characteristic UV absorption. With increasing BDAC concentration the UV-vis maximum can be shifted to higher wavelength without losing the significant enhancement in SERS experiments. In conclusion we have constructed AuNSs with more and sharper spikes, which are useful for applications in the biological window at the near infrared region.

## Conflicts of interest

There are no conflicts to declare.

## Supplementary Material

RA-009-C9RA02384D-s001
