# Supplementary material for: A simple one-step procedure to synthesise gold nanostars in concentrated aqueous surfactant solutions
Source: RSC Adv. 2019 Jul 30;9(41):23633–41. doi: 10.1039/c9ra02384d (PMC9069482; doi:10.1039/c9ra02384d)
Supplement: RA-009-C9RA02384D-s001 [file RA-009-C9RA02384D-s001.pdf]

Electronic Supplementary Material (ESI) for RSC Advances.
This journal is © The Royal Society of Chemistry 2019

Supporting Information

A simple one-step procedure to gold nanostars in concentrated aqueous surfactant solutions

Ferenc Liebig[a], Ricky Henning[a], Radwan M. Sarhan[b,c,d], Claudia Prietzel[a], Clemens N.Z. Schmitt[e], Matias Bargheer[b], Joachim Koetz[a,*]

[a]University of Potsdam,Institute for Chemistry and [b]Institute for Physics, , 14476 Potsdam, Germany

[c]Cairo University, Chemistry Department, Faculty of Science, Cairo 12613, Egypt

[d]Humboldt-Universität zu Berlin, School of Analytical Sciences Adlershof (SALSA), 10099 Berlin, Germany

[e]Max Planck Institute of Colloids and Interfaces, 14476 Potsdam, Germany

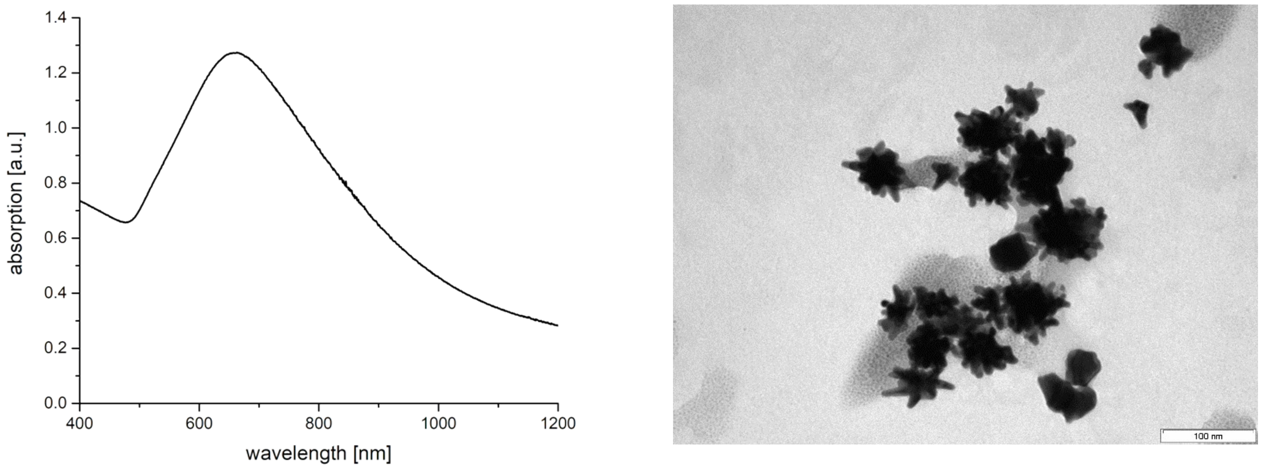

Fig. S1 Absorption spectra of SDS stabilized AuNSs and corresponding TEM micrograph.

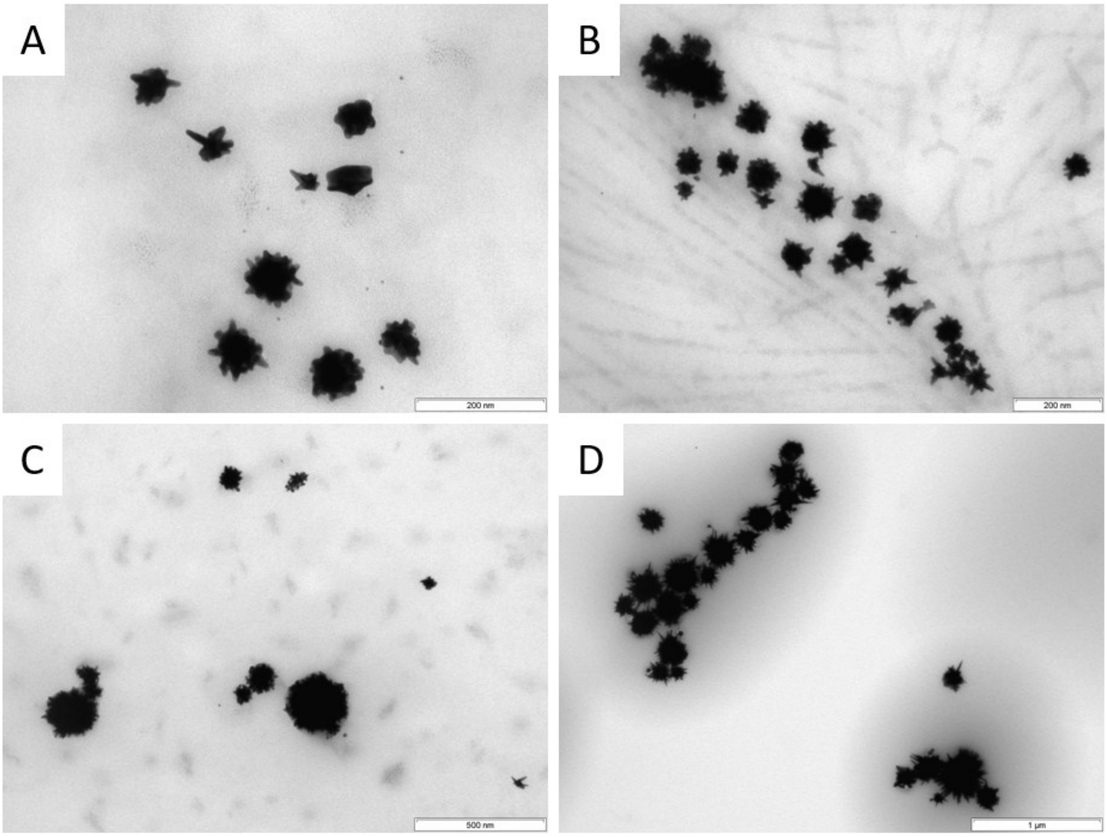

Fig. S2 TEM micrographs of AOT-BDAC stabilized AuNSs at different BDAC concentration. (A) 0.001 M, (B) 0.005 M, (C) 0.01 M, (D) 0.05 M at lower magnification.

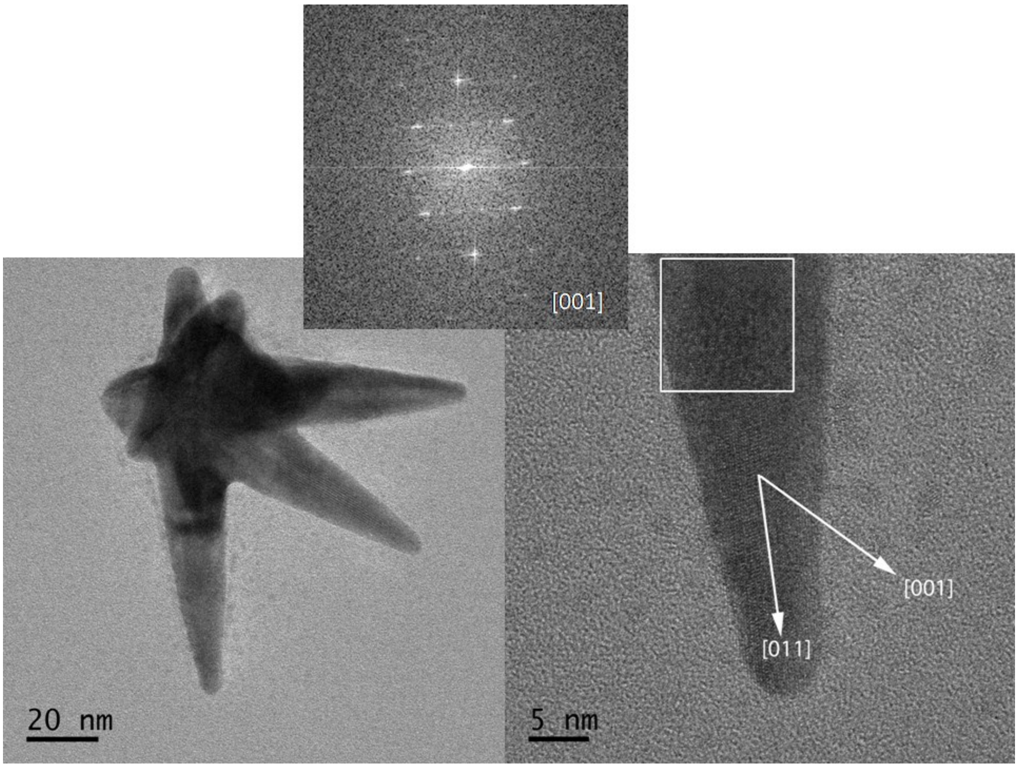

Fig. S3 Single nanostar TEM micrograph including HRTEM of the spike with corresponding FFT pattern of the marked square area.

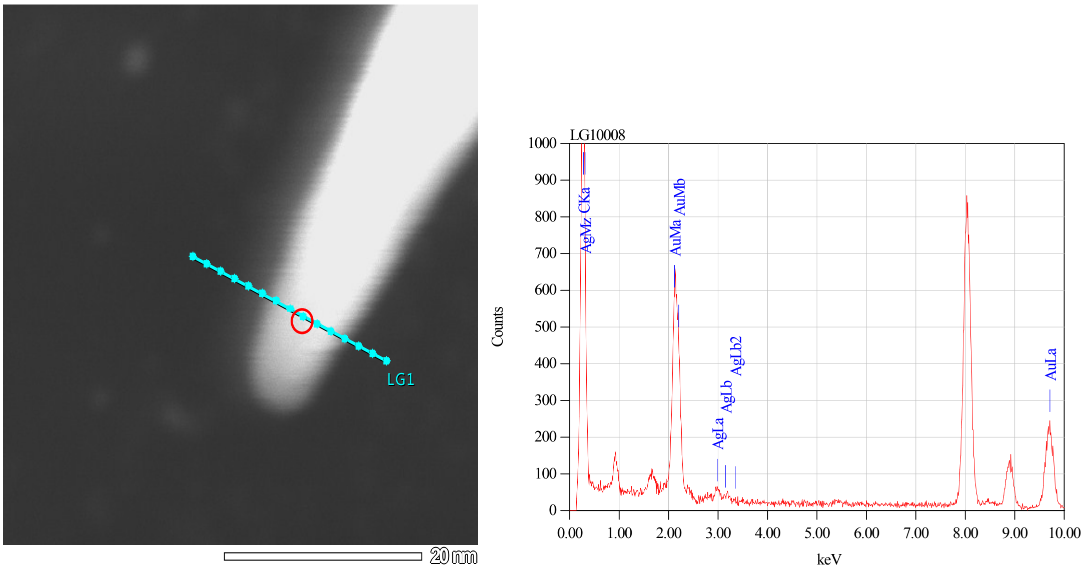

Fig. S4 Dark field TEM micrograph of an AuNS-spike with the corresponding EDX spectrum

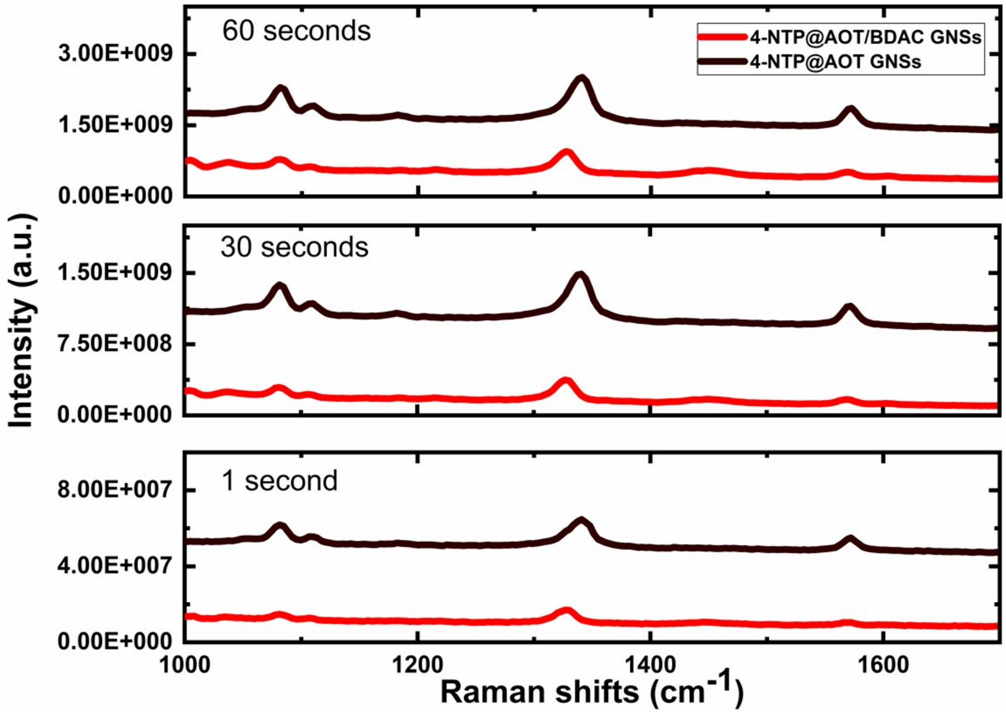

Fig. S5 Raman spectra at a low 4-NTP concentration (10^{-6} M) in dependence on the integration time
